# Metformin administration during pregnancy attenuated the long-term maternal metabolic and cognitive impairments in a mouse model of gestational diabetes

**DOI:** 10.18632/aging.103505

**Published:** 2020-07-22

**Authors:** Yalan Zhao, Xiaobo Zhou, Xue Zhao, Xinyang Yu, Andi Wang, Xuyang Chen, Hongbo Qi, Ting-Li Han, Hua Zhang, Philip N. Baker

**Affiliations:** 1 Department of Obstetrics and Gynaecology, The First Affiliated Hospital of Chongqing Medical University, Chongqing, China; 2 Chongqing Key Laboratory of Maternal and Fetal Medicine, Chongqing Medical University, Chongqing, China; 3 Department of Obstetrics, The First People's Hospital of Yunnan Province, Kunming, China; 4 Liggins Institute, University of Auckland, Auckland, New Zealand; 5 College of Medicine, Biological Sciences and Psychology, University of Leicester, Leicester, UK

**Keywords:** HPO-axis, metformin, gestational diabetes, cognitive impairment, insulin resistance

## Abstract

Background: Gestational diabetes mellitus (GDM) is a metabolic disease that can have long-term adverse effects on the cognitive function of mothers. In our study, we explored the changes in metabolic health and cognitive function in mice of middle- and old- age after exposure to GDM, and whether metformin therapy during pregnancy provided long-term benefits.

Results: Mice with GDM demonstrated significant cognitive impairment in old age, which was associated with insulin resistance. Gestational metformin therapy was shown to increase insulin sensitivity and improve cognition. The ovarian aging rate was also accelerated in mice exposed to GDM during pregnancy, which may be related to fatty acid metabolism in the ovaries.

Conclusion: Treatment with metformin during pregnancy was shown to improve fatty acid metabolism in ovarian tissues.

Method: During pregnancy, mice were fed with a high-fat diet (GDM group) or a low-fat diet (Control group), and a third group received metformin while receiving a high-fat diet (Treatment group). At 12 months old, the mice completed an oral glucose tolerance test, insulin tolerance test, Morris water maze test, female sex hormones were measured, and metabolite profiles of tissue from the ovaries, hypothalamus, and pituitary glands were analysed using gas chromatography-mass spectrometry.

## INTRODUCTION

Gestational diabetes mellitus (GDM) is a metabolic disease diagnosed when glucose intolerance is observed with onset or first occurrence during pregnancy. The global incidence of GDM is around 7 % [1]. However, in recent years, the incidence of GDM has been rising in China, with prevalence as high as 17.5 % in some areas [2]. Women who have GDM are also at an increased risk of developing other pregnancy complications including gestational hypertension, premature delivery, macrosomia, and neonatal respiratory distress syndrome [3–5]. Despite most GDM patients postpartum blood glucose levels returning to normal, these women are at a significantly higher risk of developing type 2 diabetes mellitus (T2DM) post-delivery. In women who have experienced GDM during their pregnancy, the incidence of developing diabetes was 3.7% at 9 months postpartum, 4.9 % at 15 months postpartum, 13.1 % at 5.2 years postpartum, and 18.9 % at 9 years postpartum, while the incidence of diabetes remained at 2.0 % in women without a history of GDM [6].

Women who develop GDM experience metabolic disturbances similar to that of T2DM and there seem to be equivalent pathophysiological mechanisms contributing to the complications of both medical conditions. One such complication is later life cognitive dysfunction [7–9]. It is generally accepted that persistent hyperglycemia and insulin resistance lead to brain degeneration and dysfunction, and subsequently impaired cognition. Mouse models of diabetes established by genetic modifications or fed with a high-fat diet (HFD), perform poorly on various learning and memory behavioural tests [10, 11]. Other factors contributing to cognitive dysfunction are sex-steroid hormones and aging. The main regulatory loop of the female sex hormones includes the hypothalamus, pituitary gland, and ovary (HPO axis). The hypothalamus receives information from the central nervous system and secretes a gonadotropin-releasing hormone (GnRH), which in turn stimulates the secretion of gonadotropic hormones in the pituitary gland and ultimately affects ovarian function. Hypofunction of the HPO axis is one of the important signs of female aging. Studies have found that levels of estrogen and progesterone begin to reduce as ovarian function declines [12]. Growing evidence suggests that large declines in female sex hormones that occur with menopause and aging render females more prone to diabetes, neurodegeneration, cognitive impairment, and memory disorders [13–15]. Moreover, low levels of sex steroid hormones are considered a risk factor for neurodegenerative diseases (e.g. Alzheimer’s Disease), whilst therapeutic interventions using estrogen and progesterone have been shown to be neuroprotective [15–17]. However, there is no research investigating how these risk factors will influence the long-term cognitive health of women with a history of GDM.

In this study, we aimed to investigate the associations of diabetic risk factors including hyperglycemia, insulin resistance, hormonal changes, and age, together with GDM, on the cognitive function of postpartum women during middle-old age. A GDM mouse model was established using a high-fat diet. Blood glucose levels, insulin resistance, sex hormones, and metabolic profiles of the HPO axis were measured in mice at 12 months of age. In addition, we investigated whether metformin administration during pregnancy could provide long-term protection against GDM-associated cognitive dysfunction.

## RESULTS

### Body weight, oral glucose tolerance test (OGTT), and insulin tolerance test (ITT) results of mice during pregnancy and immediately after delivery

**Figure 1:**
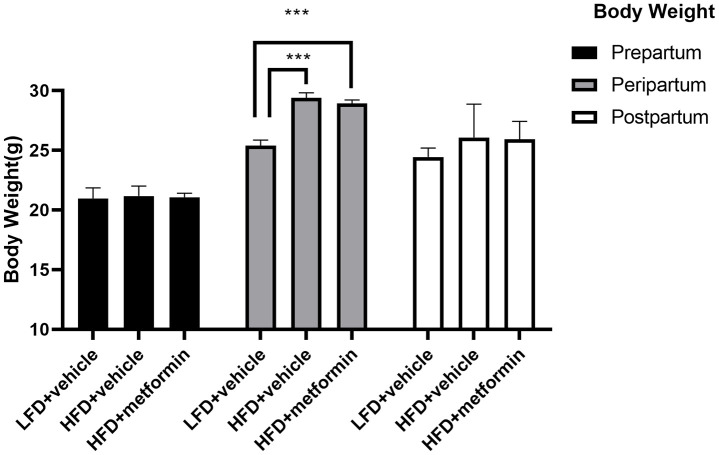
**The body weight of maternal mice before, during, and after pregnancy.** ***p-value<0.001.

**Figure 2:**
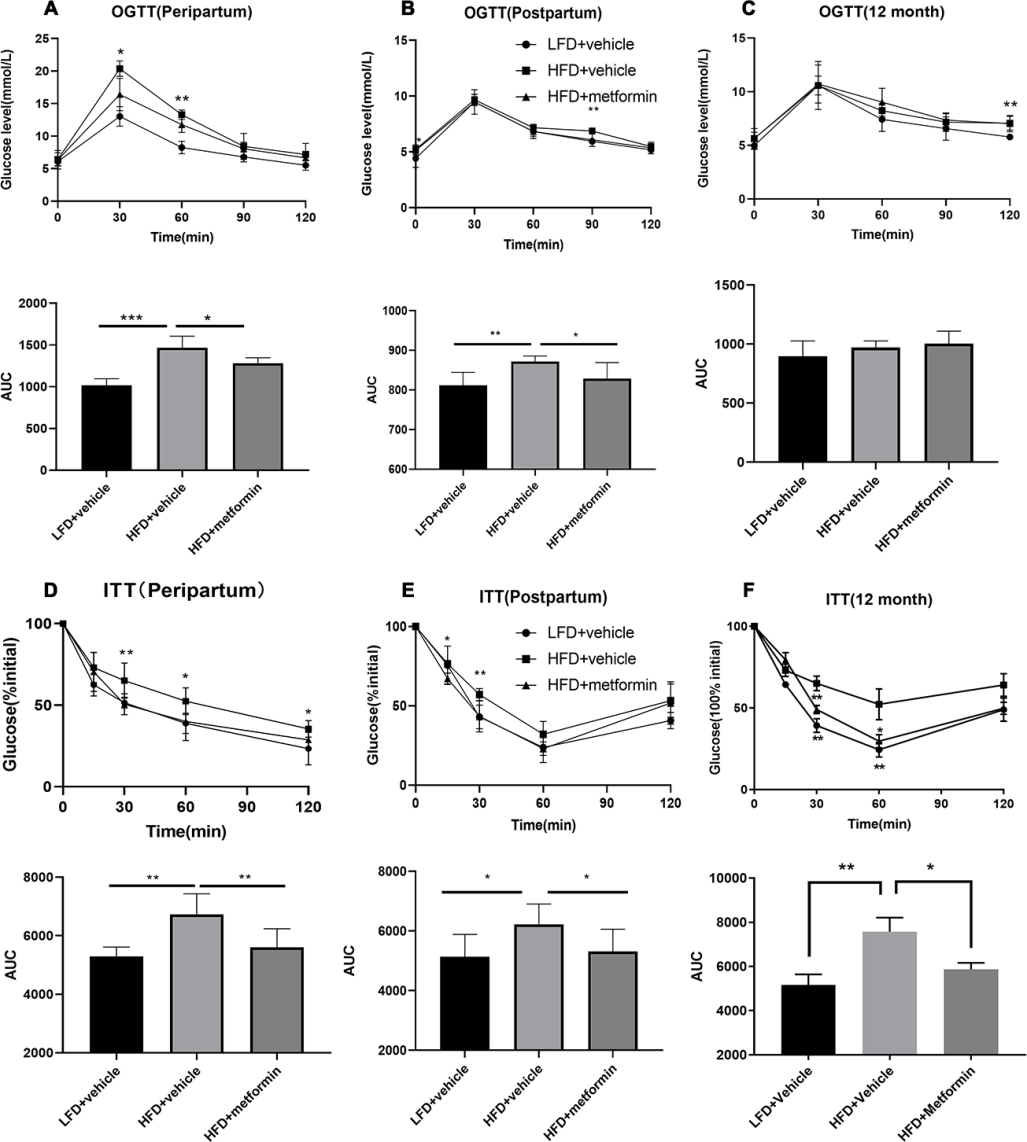
**Oral glucose tolerance test (OGTT) and insulin tolerance test (ITT) results during pregnancy (16.5 days), after delivery, and at 12 months of age.** OGTT curves for the three groups of mice and comparison of areas under the curve (AUC) during pregnancy (**A**), after delivery (**B**), and at 12 months of age (**C**). ITT curves for the three groups of mice and comparison of AUC during pregnancy (**D**), after delivery (**E**), and at 12 months of age (**F**). *p-value<0.05 versus HFD+vehicle. **p-value<0.01 versus HFD+vehicle.

As depicted in Figure 1, the body weight of maternal mice was significantly lower in the low-fat diet group (LFD+vehicle) compared to the high-fat diet group (HFD+vehicle) and metformin treatment group (HFD+metformin) during the peripartum period. At day 16.5 of pregnancy, the OGTT and ITT results exhibited a similar trend; levels at 30 min and 60 min were significantly higher in the HFD group compared to the LFD and metformin treatment groups, as were the AUCs (Figure 2A and 2D). After delivery, the HFD group had significantly higher 90 min OGTT results and both 15 min and 30 min ITT results, compared to the LFD and metformin treatment groups (Figure 2E).

### OGTT and IIT results of mice at 12 months of age

As shown in Figure 2C, the blood glucose concentrations and area under the curve (AUC) values for the OGTT were not significantly different between the three groups (F=1.624, p-value=0.230). In contrast, the blood glucose concentrations at 30 and 60 min for the ITT were significantly lower in the LFD group compared to the HFD and metformin treatment groups (Figure 2F). There was also a decreased trend in blood glucose AUC values for the ITT when comparing the LFD group to the HFD group (Figure 2F).

### Follicle-stimulating hormone (FSH), estrogen (E2), and progesterone (P) levels of mice at 12 months

**Figure 3:**
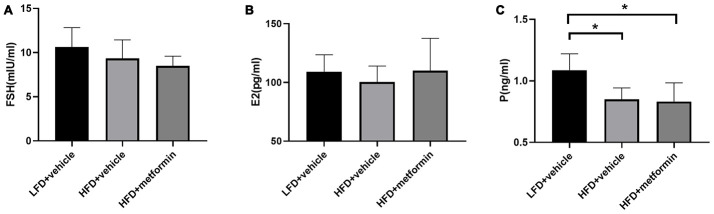
****The serum level of follicle-stimulating hormone (**A**), estrogen (**B**), and progesterone (**C**) at 12 months of age. *p-value <0.05.

There were no significant differences in serum levels of FSH or E2 across the three experimental groups (Figure 3A and 3B). However, the serum P level was significantly higher in the LFD group compared to both the HFD and metformin treatment groups (Figure 3C). These results showed that serum P levels were significantly lower in 12-month-old mice exposed to a HFD (GDM mouse model) during pregnancy and that this lower hormone level was not improved by metformin.

### Morris water maze performance

**Figure 4:**
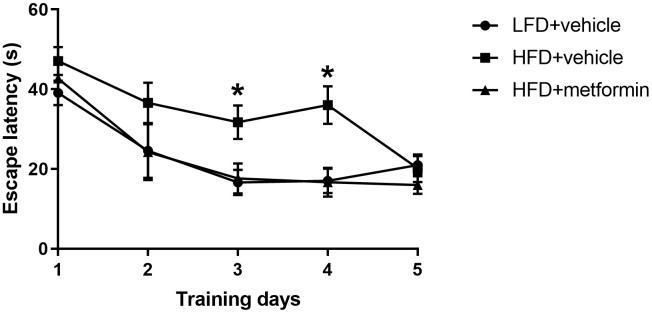
**Escape latency in the Morris water maze across five training days.** *p-value<0.05 versus HFD+vehicle.

To evaluate cognitive function of the mice at 12 months of age, we conducted the Morris water maze experiment. The results of the training day during the first five days are displayed in Figure 4. There was a significantly longer latency to escape onto the hidden platform in the HFD group when compared to both the LFD and metformin treatment groups at day 3 (p-value <0.05) and day 4 (p-value <0.05) of the training period. However, 2-way ANOVA showed that there was no statistical significance of the interaction between training days and escape latency (p-value>0.05). On experimental day one (sixth day) when the swim times and distance covered to reach the removed platform were measured (Supplementary Figure 4), the mice fed with a HFD during pregnancy exhibited a longer swim time and swimming distance (Supplementary Table 1). The HFD group also spent more time on the quadrant where the platform was previously located, as well as a greater number of times crossing the platform (Supplementary Figure 4). These findings indicate that GDM mice expressed poor spatial learning and memory capability in later life and metformin administration during pregnancy could reverse this neurocognitive deficiency.

### Metabolite profiles of the hypothalamus, pituitary gland, and ovarian tissues

**Figure 5:**
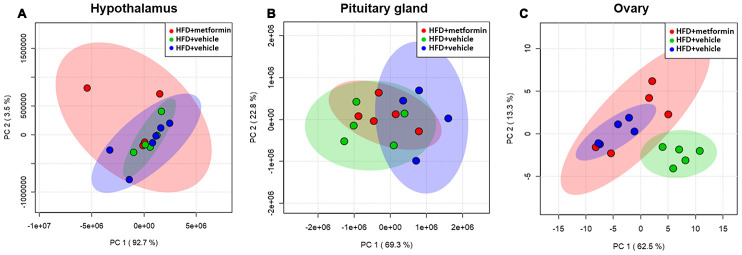
****Principal component analysis of the hypothalamus (**A**), pituitary gland (**B**), and ovary (**C**) collected from maternal mice at 12 months of age. The color legends of experimental mice groups are listed as follows; Red color indicates maternal mice fed with a high-fat diet and metformin during pregnancy (HFD+metformin); Green color indicates maternal mice fed with a low-fat diet during pregnancy (LFD); Blue color indicates maternal mice fed with a high-fat diet during pregnancy (HFD).

**Figure 6:**
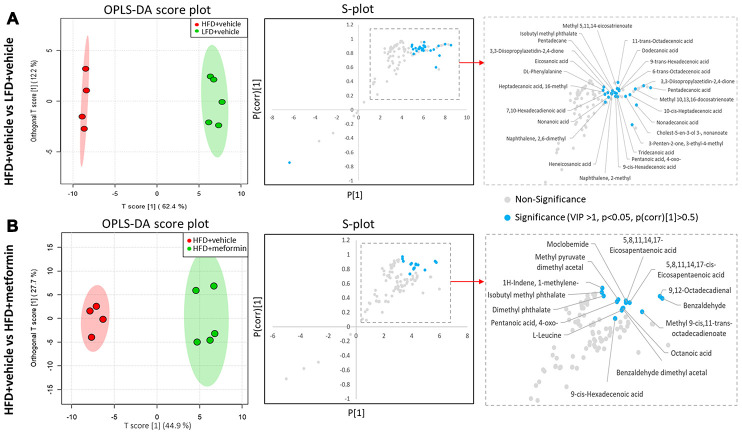
**Identification of significant metabolites in the ovary.** (**A**) OPLS-DA and S-plot were used to detect metabolites that discriminated between HFD+vehicle (red) and LFD+vehicle (green). (**B**) OPLS-DA and S-plot were used to detect metabolites that discriminated between HFD+vehicle (red) and HFD+metformin (green). In S-plot, the blue circles indicate significant metabolites with a VIP of > 1, p-value <0.05, and P(corr) >0.5.

**Figure 7:**
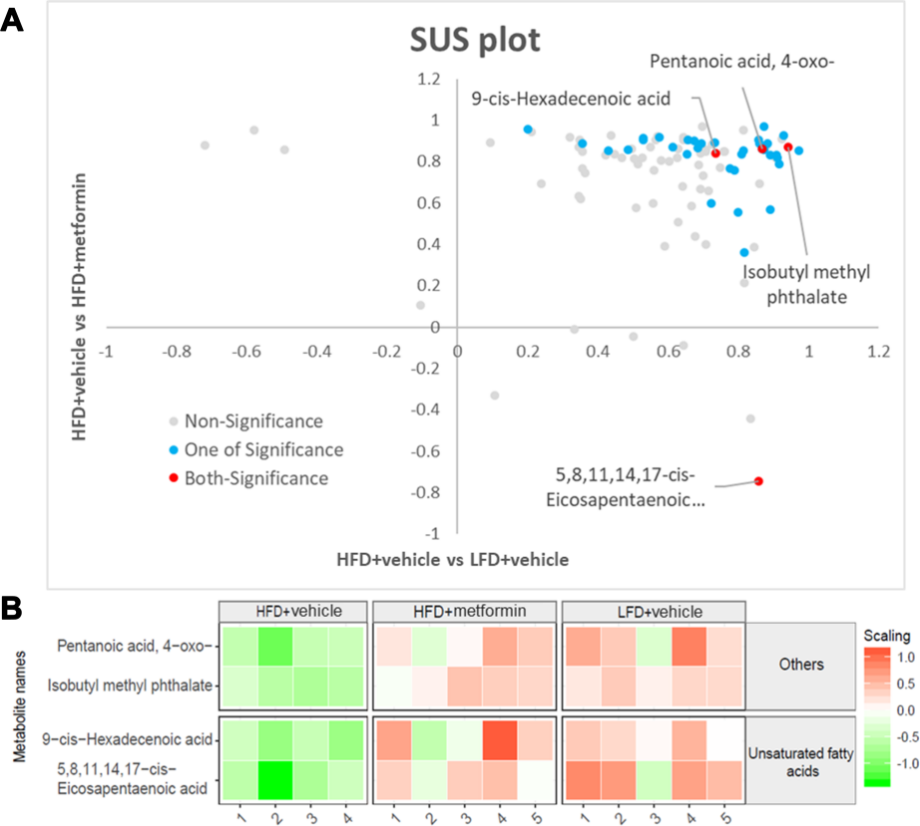
**Significant ovary metabolites common to the LFD and metformin treatment groups.** (**A**) SUS-plot. The red circles represent the metabolites common to both OPLS-DA models (LFD vs HFD and HFD+metformin vs HFD). The blue circles represent metabolites in at least one of the two OPLS-DA models. (**B**) The heatmap illustrates the levels of the final shortlisted metabolites in each group. The relative concentrations of metabolites were log_2_ transformed and Pareto scaled. Red color indicates a higher level, while green color indicates a lower level.

A total of 125 compounds were identified in the hypothalamus, pituitary gland and ovary. The PCA demonstrated no obvious separation of the hypothalamus and pituitary gland tissue between the LFD, HFD, and metformin treatment groups (Figure 5A and 5B). The metformin treatment group and LFD group were clustered together and separated away from the HFD group in the ovary metabolite profile (PC1 and PC2 explained 62.5% and 13.3% of variance, respectively; Figure 5C). We then utilised OPLS-DA and S-plot to screen for statistically important metabolites leading the discrimination of each of the LFD and metformin treatment groups from the HFD group. As demonstrated in Figure 6, both OPLS-DA models (First model, LFD+vehicle vs HFD+vehicle; Second model, HFD+metformin vs HFD+ vehicle) yielded good class separation and statistical validation (R2 = 0.89, Q2 = 0.87; R2 = 0.82, Q2 = 0.78, respectively). By combining VIP scores of the OPLS-DA (VIP > 1), Student’s-T test (p-value >0.05), and covariance of the S-plot (p (corr)| > 0.5), a shortlist of significant metabolites was generated. A list of 25 and 15 metabolites passed the selection criteria for the LFD+vehicle vs HFD+vehicle and HFD+metformin vs HFD+vehicle, respectively (Figure 6A and 6B). Finally, a SUS-plot was constructed to identify the significant metabolites that were shared and unique in the two OPLS-DA models (Figure 7A). Four metabolites: 4-oxo pentanoic acid, 9-cis-hexadecenoic acid, isobutyl methyl phthalate, and 5,8,11,14,1,7-cis-eicosapentaenoic acid were significant in both models. The heatmap in Figure 7B shows that all of the shortlisted metabolites were in lower concentrations in the HFD group compared to the LFD and metformin treatment groups. The changes in these metabolites between the HFD group and the metformin treatment group may represent the effect of metformin treatment on the ovaries of 12 month old mice exposed to GDM during pregnancy.

## DISCUSSION

In this study, we investigated how risk factors such as hyperglycemia, insulin resistance, aging, and abnormal levels of female sex hormones contribute to the acceleration of cognitive decline in mice of middle-old age who had previously had GDM. Results of the Morris water maze test showed that compared to the control group, the cognitive function of mice in the GDM group was significantly reduced after entering middle-old age. We also observed impaired insulin tolerance and greater metabolic changes downstream of the HPO axis at 12 months of age. Treatment with metformin during pregnancy significantly improved the long-term cognitive function of mice exposed to GDM during pregnancy.

We have successfully established a mouse model that resembles the pathophysiological state of GDM; including having a higher body weight and confirmed hyperglycemia (Figures 1 and 2). No difference in glucose tolerance was observed between GDM and control mice at 12 months (Figure 2C). Hyperglycemia in GDM only occurred temporarily during gestation and blood glucose levels gradually returned to normal post-delivery. Thus, hyperglycemia in later life did not seem to be responsible for the GDM-related cognitive impairment. Moreover, of the hormones analysed, only progesterone levels were significantly reduced in the GDM and metformin treatment groups at 12 months (Figure 3). Progesterone typically declines earlier than estrogen in the first stages of perimenopause. Studies have suggested that cognitive impairment related to the perimenopausal period is related to the reduction of estrogen [18, 19]. Estrogen is thought to be involved in maintaining higher cognitive functions by inducing spinogenesis and synaptogenesis in the prefrontal cortex and hippocampus, via estrogen receptors [19]. A plethora of evidence has been presented which shows that estrogen loss resulting from menopause or the surgical removal of an ovary/ovaries, can accelerate age-related cognitive decline. Estrogen replacement therapy has been shown to improve the cognitive function of these women [20]. Reductions in progesterone without reductions in estrogen merely represent early-stage functional decline of the ovaries, as part of the natural process of early perimenopause. Therefore, our results suggest that hyperglycemia, female sex hormones, and aging may not be the key factors leading to the cognitive impairments observed in the GDM group in later life.

Although hyperglycemia observed during GDM resolves postpartum, insulin resistance persists [21, 22]. Insulin resistance appears to be the most likely mediator of the cognitive impairment observed in later life, after exposure to GDM during pregnancy. Our results indicate that the GDM group had significantly reduced insulin sensitivity at 12 months of age (Figure 2F). Therefore, a mother who develops GDM manifests similar metabolic disturbances to T2DM, which is characterized by insulin resistance and insulin signalling deficiency [23, 24]. Many animal studies have emphasized the direct association between insulin resistance and cognitive dysfunction [25, 26]. Studies using mouse models of T2DM have revealed that hippocampal insulin resistance leads to neuroplasticity impairment [27–29]. HFD-induced models of diabetes have observed a reduction in cell proliferation and neurogenesis in the dentate gyrus of the hippocampus [30]. Furthermore, peripheral insulin resistance can directly contribute to brain insulin resistance by compromising transport of insulin into the central nervous system [31]. The restoration of insulin signalling activities in the hippocampus has been shown to alleviate the cognitive decrements observed in a T2DM mouse model [32]. These studies show that insulin resistance causes structural and functional abnormalities in the brain. Therefore, persistent insulin resistance appears to be an important factor in the long-term reduction in cognitive function observed following GDM. In addition, recent studies have shown aberrant gut microbiota can result in the development of cognitive dysfunction and T2DM in diabetic mouse models [33, 34]. The findings from these studies suggest that the gut microbiota could also influence host insulin sensitivity and cognitive function.

Despite the sex hormones remaining at a normal physiological level in the GDM group at 12 months of age, a sex hormone regulation and feedback system “HPO axis” was investigated to see whether it was adversely affected by exposure to GDM. A cascade amplification effect on the downstream regulation of HPO was observed in the early perimenopausal period. In particular, substantial metabolic changes were detected in ovarian tissues of both normal and treatment groups when compared to GDM groups (Figure 5). Pentanoic acid, 9-cis-hexadecenoic acid, and isobutyl methyl phthalate were significantly lower in ovarian tissue from the GDM group (Figure 7). 9-cis-hexadecenoic acid (palmitoleic acid) is a common unsaturated fatty acid in the ovary that regulates gonadotropin-stimulated progesterone biosynthesis in granulosa cells [35]. 9-cis-hexadecenoic acid can also synergistically stimulate cell proliferation via IGF-1 [36]. Liu et al (2001) [37] has previously reported that pentanoic acid has a potent effect on stimulating protein synthesis without accelerating cell apoptosis in a rodent ovarian cell culture. Since these fatty acids are involved in protein biosynthesis, anti-apoptosis, hormone production, and cell proliferation, lower levels of these metabolites may accelerate the aging process in the ovaries of women with a history of GDM. The process of female reproductive aging is believed to be predominated by the age-associated decline in ovarian function. The number of follicles in the ovary are diminished and oocyte quality declines as chronological age increases. This leads to a reduction in ovarian hormones and subsequently compromises the negative feedback from the ovarian sex-steroids to the hypothalamic-pituitary axis [38]. Because maternal mice in our study were sampled at the premenopausal stage, the ovarian feedback status had not diminished enough to dysregulate the upstream components of the HPO axis. Based on these findings, we postulate that GDM manifestation results in a negative metabolic influence on the downstream aspects of the HPO axis and thereby accelerates postpartum ovarian aging.

To investigate the therapeutic effect of metformin, a drug known to increase peripheral target cell insulin sensitivity, mice with GDM were administrated metformin during pregnancy. Our results demonstrated that insulin sensitivity was returned to a normal level in the metformin treatment group, as shown in Figure 4C. Metformin has been reported to reduce insulin resistance by activating AMPK and SIRT1 to inhibit hepatic gluconeogenesis, suppress lipid biosynthesis, and enhance glucose metabolism, thus decreasing circulating glucose and lipid levels [39–41]. Metformin can also reduce insulin resistance by downregulating chemerin and suppressing endoplasmic reticulum stress in adipose tissue and liver cells [39, 42]. A recent human gut metagenomic study indicated that metformin treatment can improve insulin sensitivity by favoring gut *Escherichia* species. In doing so, the authors observed an increase in microbial short-chain fatty acid production, attenuation of intestinal lipid absorption, and reduced lipopolysaccharide (LPS)-induced inflammation [43]. Importantly, we also found that GDM mice administrated metformin during pregnancy had spatial learning and memory capability scores equivalent to normal mice at 12 months of age (Figure 4). Although clinical studies have shown that the administration of metformin during pregnancy can effectively control blood glucose and improve insulin sensitivity in GDM [44–46], our findings highlight the long-term protective effects of metformin administration on cognitive health. Evidence strongly supports the role of insulin resistance in cognitive decline and it has been suggested that an insulin sensitizer may prevent against cognitive decline in pre-diabetic and diabetic patients [10, 47–48]. Metformin’s proposed mechanisms of action on neurons and microglia include AMPK-associated neural proliferation, differentiation, self-renewal, autophagy, and energy homeostasis [10, 49–57]. Metformin inhibits inflammation by suppressing NF-κB [58] as well as promoting glucose consumption, lactate production, and reducing oxidative phosphorylation, thereby favouring glycolytic metabolism in astrocytes and microglia [59–61]. Based on these findings, we speculate that metformin therapy during GDM pregnancy may reverse the long-term maternal cognitive impairments in middle-old age by attenuating persistent postpartum insulin resistance. Despite metformin treatment during pregnancy not raising progesterone levels in the GDM group, treatment with metformin seemed to protect the downstream aspects of the HPO axis, including ovarian tissue metabolism. These findings could be explained by metformin’s ability to reduce blood glucose levels by minimizing the absorption of glucose in the intestine and inhibiting gluconeogenesis in the liver, without stimulating the secretion of insulin [62–64].

**Figure 8:**
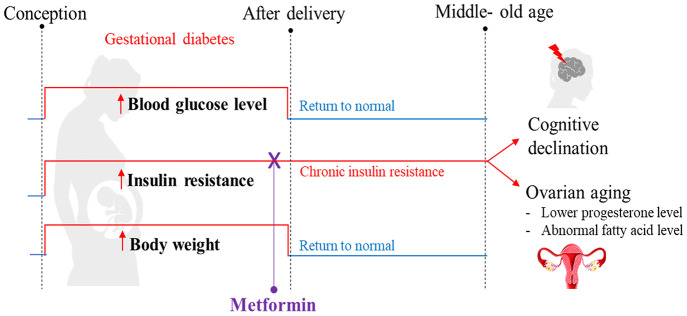
**Summary of the proposed mechanism explaining how gestational diabetes mellitus (GDM) may lead to cognitive impairment and ovarian aging later in maternal life.** Our high-fat diet (HFD) mouse model exhibited the pathophysiological phenotype that resembles GDM, including hyperglycemia, being overweight, and experiencing insulin resistance during pregnancy. After delivery, all mice were reverted back to a standard diet. In the postpartum period, the blood glucose level and body weight returned to normal, but insulin resistance persisted and reduced cognitive function was observed at 12 months of age. A reduced progesterone level was also observed; an early indication of perimenopause. Through metabolome profiling of the hypothalamus, pituitary gland, and ovarian (HPO) axis, a downstream dysregulation of the HPO axis was revealed. In particular, ovarian fatty acid levels were reduced. All these adverse outcomes were prevented when insulin resistance during pregnancy was treated with metformin. These phenotypes were not directly associated with hyperglycemia or high-fat diet during gestation, nor differences in bodyweight after pregnancy. Based on these observations, we hypothesize that persistent insulin resistance postpartum is the primary cause of GDM-related cognitive impairment and accelerated ovarian decline.

The results of our study demonstrated that despite postpartum weight, diet, and blood glucose returning to a normal status after GDM, insulin resistance persisted. We propose that prolonged insulin resistance is likely responsible for the adverse effects on long-term endocrine and cognitive functions (Figure 8). The potential clinical translation of this finding is that medical professionals should monitor women with a history of GDM, even if their weight and blood glucose return to normal after delivery. In particular, their insulin tolerance should be regularly monitored. If insulin resistance remains persistent, it should be corrected in order to protect the endocrine and neurocognitive functions of women. More importantly, persistent postpartum insulin resistance may be minimized or avoided in GDM mothers by administrating metformin during pregnancy. Future research should include mechanistic studies such as gene knockout and cell models to test whether persistent postpartum insulin resistance is the primary cause of cognitive impairment and ovarian aging. Additional studies are also needed to test whether a higher dose of metformin supplementation during pregnancy or extending its treatment time to the postpartum period could improve maternal hormonal levels.

In conclusion, the cognitive function of mice exposed to GDM was significantly impaired compared to controls after entering middle- old age. Our findings suggest that the cognitive impairments were mainly related to persistent insulin resistance after delivery. Treatment of GDM with metformin during pregnancy significantly improved postpartum insulin resistance and was protective of long-term cognitive health. In addition, GDM altered the long-term metabolism of the ovary and thus accelerated the chronological aging process. Although metformin treatment of GDM during pregnancy improved the metabolite pattern of the HPO axis, whether it can further affect the function of the HPO axis remains to be investigated.

## MATERIALS AND METHODS

### Overall experimental design

**Figure 9:**
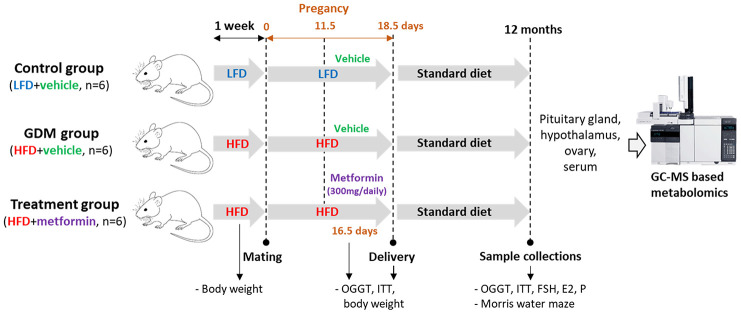
Overall experimental design.

Eighteen C57BL/6 mice were randomly divided into three groups: control (low-fat diet (LFD) + vehicle, n=6), GDM (high-fat diet (HFD) + vehicle, n=6) and metformin treatment (HFD+metformin, n=6). There was no metformin treatment for the LFD group because our pilot study showed that metformin treatment did not change the blood glucose level or insulin resistance in the mice fed with a LFD during pregnancy (Supplementary Figure 1). The control mice were fed a low-fat diet (Research Diets AIN-93G, consisting of 20.3 % protein, 63.9 % carbohydrate, and 15.8 % fat) for one week prior to mating and throughout pregnancy (18.5 days), while both GDM and treatment groups were fed a high-fat diet (Research Diets D12451, consisting of 20% protein, 35 % carbohydrate, and 45 % fat). All mice were given free access to 100 grams of fresh diet and 250 ml of fresh water daily per cage (Five mice per cage). From 11.5 to 17.5 days of pregnancy, the treatment group received a 300 mg dose of metformin solution each day via oral gavage, whilst the control and GDM group received the same dose of a vehicle (phosphate buffer solution). After delivery, all maternal mice were reversed back to normal chow (Research Diet 1022, consisting of 18% protein, 78% carbohydrate, and 4 % fat) until the age of 12 months. At 12 months of age, the mice performed the Morris water maze experiment, and underwent an oral glucose tolerance test (OGTT) and insulin tolerance test (ITT). In addition, the body weight of the mice was measured prepartum, peripartum and postpartum. After the mice were sacrificed, 200 μl blood was collected by cardiac puncture. The blood was centrifuged at 1300 g for 10 min at 4 °C in order to obtain serum for hormone measurements such as follicle-stimulating hormone (FSH), estrogen, and progesterone. Furthermore, the ovaries, hypothalamus and pituitary gland were collected and stored in a -80 °C freezer until gas chromatography-mass spectrometry (GC-MS) based metabolomics was performed (Figure 9). The handling of animals in this research was in accordance with the guidelines approved by the ethics committee of the First Affiliated Hospital of Chongqing Medical University (Ethics number 2016-41). All efforts were taken to minimize the number of mice sacrificed and any potential suffering.

### Oral glucose tolerance test (OGTT) and insulin tolerance test (ITT)

An OGTT and ITT were performed during pregnancy at 16.5 days, immediately after delivery, and at 12 months of age. OGTT was performed by first fasting mice for six hours then administering glucose (2g/kg body weight) via gavage. Blood samples were collected at 0, 30, 60, 90, and 120 minutes from the tail vein, and blood glucose concentration was measured using a glucometer (Nova StatStrip Xpress; Nova Biomedical, Waltham, UK). To perform ITT, the mice were fasted for 6 hours and then insulin (2.5 U/kg body weight) was administered via an intraperitoneal injection. Blood samples were collected at 0, 15, 30, 60, and 120 minutes from the tail vein, and blood glucose concentration was measured using a glucometer.

### Morris water maze experiment

The Morris water maze experiment was conducted in a constant room temperature swimming pool with a white inner wall which had a diameter of 120 cm, a height of 60 cm, and a data collection and analysis system. The entire pool was divided into four quadrants by four equally spaced points (indicated by different patterns). The hidden platform (10 cm in diameter and 24 cm in height) was placed 0.5 cm below the water surface. The animals had five continuous days of maze training where they were placed in the middle of each quadrant of the maze. During the training days, the mice were guided to the hidden platform if they failed to locate the platform within one minute and stayed on the hidden platform for 10 s. During the experiment day (sixth day), the hidden platform was eliminated, and mice were swimming in the maze for 60 s. This experiment was repeated four times a day.

### Measurement of serum follicle-stimulating hormone (FSH), estrogen (E2), and progesterone (P)

Serum levels of FSH, E2, and P were analysed using ELISA kits (cat. no. CSB_E06871m; CSB_E05109m; CSB_E05104m). All analyses were performed in strict accordance with the manufacturer's protocols.

### Metabolite extraction and derivatization

An approximately 10 mg sample of the ovary, hypothalamus, and pituitary gland from each animal were weighed at 4 °C. Metabolites were extracted from the samples using 2 mL of methanol/toluene 4:1 v/v solution containing two internal standards: nonadecanoic acid (20 μg/mL) and tridecanoic acid (20 μg/mL). 200 μL of acetyl chloride (Adamas Reagent Co.) was added to each sample, followed by a 1 min vortex. The tubes were then incubated at 100 °C for 1 h. After cooling in tap water, 5 mL of an aqueous solution of 6% potassium carbonate (Adamas Reagent Co.) was added into each tube. After vortexing for 10 s and centrifuging at 2000 rpm for 10 min at room temperature, the upper toluene phase was extracted for GC-MS analysis. Negative controls and quality control (QC) samples were prepared by replicating the sample preparation using empty tubes and a pooled biological sample respectively.

### Gas chromatography-mass spectrometry (GCMS) analysis

The derivatized biospecimens were analysed using an Agilent 7890B Gas Chromatograph linked to an Agilent 5977A Mass Spectrometer. A RESTEK Rtx®-2330 column (90% biscyanopropyl/10% phenylcyano propylpolysiloxane, 100 m, 0.25 mm ID, 0.2 um df) was installed to separate derivatised metabolites. The sample injection, inlet mode, oven temperature, and mass spectrometry parameters were operated according to Han et al. (2012) and Smart et al. (2010). The GC-MS chromatographic peaks were extracted, deconvoluted and identified using AMDIS and Agilent ChemStation. The peaks were identified based on two criteria; >85% match to the fatty acid library spectra and within a 30-second window of the library chromatographic retention time using the in-house lipid library and NIST library (https://www.nist.gov/nist-research-library). The relative concentrations of metabolites were quantified via our in-house R based script (MassOmics) that uses the most abundant ion fragments within an appropriate retention time.

### Data normalization and statistical analysis

Metabolite levels were first normalized either by the nonadecanoic acid or tridecanoic acid internal standard, determined based on their correlation with metabolites in the QC samples. Subsequently, median centering was performed using nine QC samples to correct for batch variation. Then, correction was applied using blank samples to remove contaminants and any carryover from identified metabolites. The tissue weights were accounted for, to eliminate the volume difference among samples. Prior to multivariate statistical analysis, the metabolite profiles of the HPO axis were log transformed and Pareto scaled since this combined scaling strategy resulted in a normal distribution of our dataset. Principal component analysis (PCA), orthogonal partial least squares discriminant analysis (OPLS-DA), variable importance projection (VIP) scores, one-way ANOVA, Turkey HSD, S-plots, and SUS-plots were performed using Metaboanalyst (https://www.metaboanalyst.ca/) and our in-house R scripts. A power analysis for the two-sample t-test was performed using the “pwr.t.test” command available in the R package “pwr2” [65]. The power analysis was performed based on the result of Zhu et al (2018), which utilized a HFD to establish a GDM mouse model, to evaluate the effect of maternal obesity on cognitive development of the offspring. Our power calculation showed that a minimum sample size of six mice per group provided 80% power with an alpha value less than 0.05 in order to have 95% confidence of a true difference between HFD and LFD groups for both ITT and OGTT (Supplementary Figures 2 and 3).

## Supplementary Material

Supplementary Figures

Supplementary Table 1
