# Supplementary material for: Metformin administration during pregnancy attenuated the long-term maternal metabolic and cognitive impairments in a mouse model of gestational diabetes
Source: Aging (Albany NY). 2020 Jul 22;12(14):14019–36. doi: 10.18632/aging.103505 (PMC7425475; doi:10.18632/aging.103505)
Supplement: Supplementary Figures [file aging-12-103505-s002..pdf]

SUPPLEMENTARY FIGURES

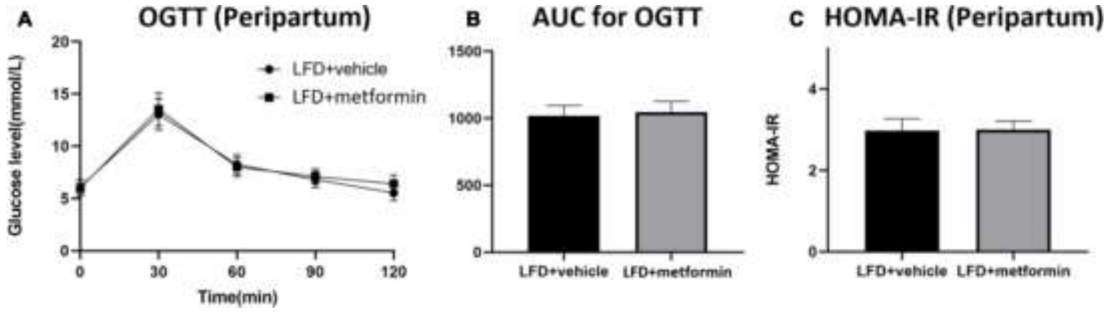

Supplementary Figure 1. Oral glucose tolerance test (OGTT) and homeostasis model assessment of insulin resistance (HOMA-IR) during pregnancy. OGTT curves between the LFD+vehicle and LFD+metformin treatment groups (**A**) and comparison of areas under the curve (AUC) at 16.5 gestational days (**B**). HOMA-IR results between the LFD+vehicle and LFD+metformin treatment groups at 18.5 gestational days (**C**).

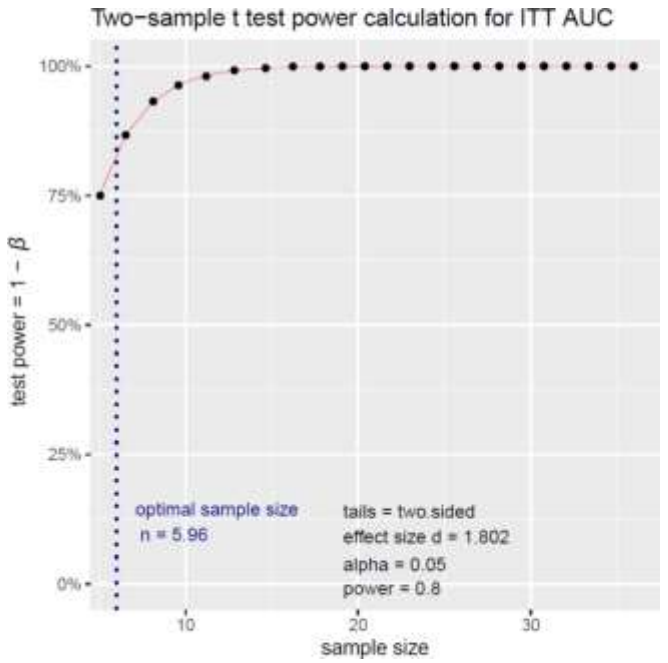

Supplementary Figure 2. Power analysis of ITT between HFD and LFD mice at peripartum, based on data from Zhu et al (2018). The curve displays the relationship between changes of power (y-axis) along with changes in sample size (x-axis). The blue vertical line indicates sample size required to have 80% power with an alpha value less than 0.05 for a given mean difference between two groups using a two-sided Student’s T test. n= number. AUC = area under the curve.

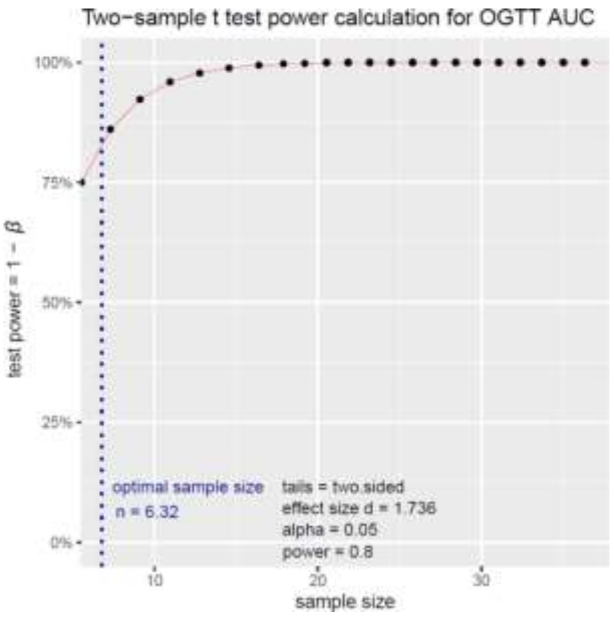

Supplementary Figure 3. Power analysis of OGTT between HFD and LFD mice at peripartum, based on data from Zhu et al (2018). The curve displays the relationship between changes of power (y-axis) along with changes in sample size (x-axis). The blue vertical line indicates sample size required to have 80% power with an alpha value less than 0.05 for a given mean difference between two groups using a two-sided Student's T test. n= number. AUC = area under the curve.

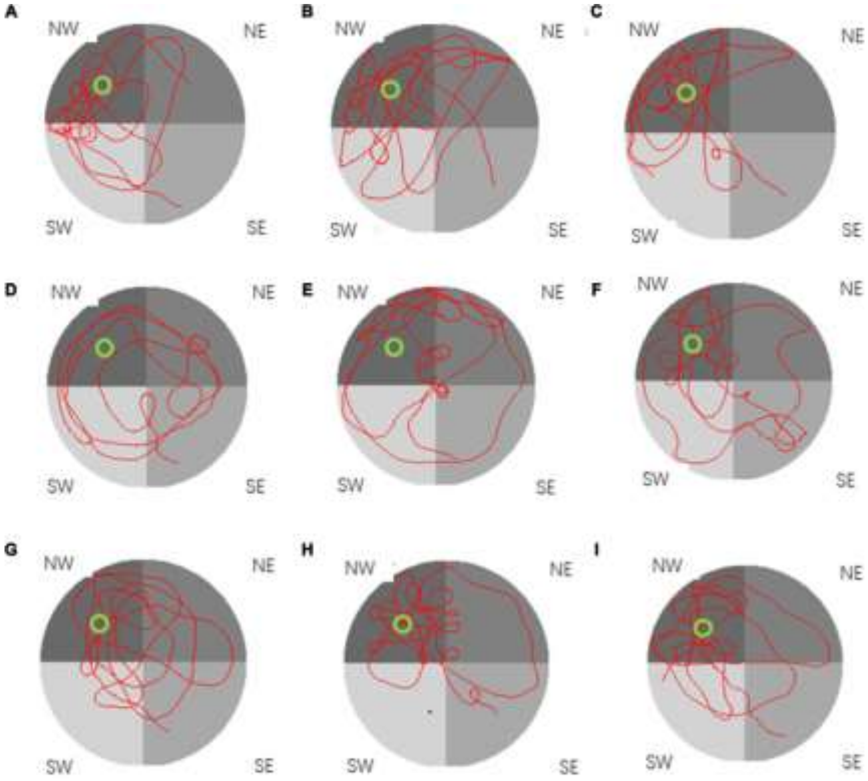

Supplementary Figure 4. Representative swimming tracks in the Morris water maze. Swimming tracks of mice in the control group (**A**–**C**). Swimming tracks of mice in the GDM group (**D**–**F**). Swimming tracks of mice in the treatment group (**G**–**I**). The circle represents the area of the water maze, divided into four quadrants, the green circle represents the location of the original platform, and the red line represents the swimming route of the mouse.
