# Supplementary material for: Metformin administration during pregnancy attenuated the long-term maternal metabolic and cognitive impairments in a mouse model of gestational diabetes
Source: Aging (Albany NY). 2020 Jul 22;12(14):14019–36. doi: 10.18632/aging.103505 (PMC7425475; doi:10.18632/aging.103505)
Supplement: Supplementary Table 1 [file aging-12-103505-s001..pdf]

SUPPLEMENTARY TABLE

Supplementary Table 1. Morris water maze results.

	Control (n=7)	GDM (n=6)	Treatment (n=6)	F	p-value
Time (s)	4.0±1.1*	1.3±1.5	2.3±0.8	7.903	0.005
Time (s in %)	54.0±7.3*	35.3±14.7	54.1±8.7*	6.100	0.012
Distance (cm in %)	51.2±8.3**	34.8±13.3	51.3±8.2**	5.209	0.019

Time (s in %) is calculated as swimming time in the quadrant of the original platform/total swimming time*100. Distance (cm in %) is calculated as swimming distance in the quadrant of the original platform/total swimming distance*100. F represents the statistic of one-way Anova, *p-value<0.05 versus HFD+Vehicle, **p-value<0.01 versus HFD+Vehicle
